# Mental health among people with a migration background in Belgium over the past 20 years: how has the situation evolved?

**DOI:** 10.1186/s13690-023-01187-z

**Published:** 2023-09-28

**Authors:** Camille Duveau, Pierre Smith, Vincent Lorant

**Affiliations:** 1 Faculty of Public Health, Institute of Health and Society (IRSS), Université catholique de Louvain, B1.31.15, Brussels, 1200 Belgium; 2 Department of Epidemiology and Public Health, Sciensano, Brussels, Belgium

**Keywords:** Mental health, Migrants, Ethnic minorities, Depression, Psychological distress

## Abstract

**Background:**

Poor mental health is highly stigmatized and stereotyped, even more when it comes to migrant and ethnic minority groups (MEM). Belgium, which has a long history of immigration, is a good case study for analysing how the prevalence of mental illness (MI) has evolved over time and how such evolution had differed between MEM. This paper seeks to explore the prevalence of MI and potential inequalities among MEM compared to native Belgians between 1997 and 2018, shedding light on this important issue.

**Methods:**

The data set is composed of the six cross-sectional waves of the Belgian Health Interview Survey from 1997 to 2018. The 12-item General Health Questionnaire was used to assess the average level of mental health and the prevalence of MI (score ≥ 4) among five major MEM groups in Belgium (Belgian, Moroccan, Turkish, European migrants, and non-European migrants). Multivariate logistic and linear regression models were used to assess the likelihood of having a MI in the different MEM groups and survey years. The minimal clinically important difference (MID) was also calculated for the severity of MI.

**Results:**

After controlling for socioeconomic status, the average marginal effect indicated a decrease in mental health among Moroccans and Turks in Belgium between 1997 and 2018, compared to Belgians. This result was confirmed by the Chi²-test, which showed that Turkish (χ²=17.75, p < 0.001) and Moroccans respondents (χ²=4.19, p < 0.04) had a higher overall level of mental distress than Belgians. Furthermore, in 2018, even after adjusting for age, sex and education level, having a mother born in a non-EU country increased the risk of mental illness.

**Conclusions:**

Mental health inequalities between migrant and ethnic groups are on the rise in Belgium. To address this issue, particular attention should be given to the Moroccan and Turkish background populations. Specific interventions and policies must be implemented to prevent the increase of psychological distress among migrants and ethnic minorities, on the one hand, and ensure that high-quality mental health care is accessible to all, regardless of ethnicity, on the other hand. Additionally, we recommend that future research on ethnic mental healthcare includes better data collection on the country of birth of respondents and their parents.

**Table:** 

**Text box 1. Contributions to the literature**
• What is known
Migrants and ethnic minorities frequently experience a higher incidence of mental health disorders, including depression, compared to the host country populations across numerous European countries, especially in Belgium.
• What does the study add
This research contributes to a comprehensive understanding of the evolution of mental health vulnerabilities within migrant and ethnic minority (MEM) communities, contrasting these patterns with the Belgian populations.
• What the implications are for clinical practice, public health and/or research.
Informed by the findings of this study, we can provide the trajectory of mental health risks among five significant MEM groups in Belgium, spanning the time frame from 1997 to 2018. This insight holds valuable implications for clinical practitioners, public health initiatives, and ongoing research endeavours.

## Background

People who were born abroad and people with parents born abroad, often referred to as migrants and ethnic minority groups (MEM), are a vulnerable group to psychological distress [1, 2]. Yet, there is little literature addressing the longitudinal evolution of their psychological distress in Belgium. One issue is that previous studies have shown that MEM populations were at a higher risk of mental health disorders, particularly MEM from the second generation [1, 3]. However, it remains unclear whether and how this risk has evolved over time. In Belgium, MEM may have faced additional risk factors related to lower socioeconomic status, social exclusion, traumatic experiences, discriminatory practices, and cultural adaptation which could increase their risk of mental illness (MI) [4–8]. Additionally, MEM encounter various challenges such as language barriers, unemployment, and social isolation that negatively impact their integration into the host country [9]. Despite these challenges, MEM are not receiving equal mental health care or are unable to access quality mental health care is not acceptable, as it is an essential human right [10–12].

Turkish and Moroccan immigrants form one of the largest established groups in Belgium, and migrants make up 20% of the Belgian population and up to 40% in Brussels, the capital [13]. In 2007, Levecque and colleagues found that persons of Turkish or Moroccan origin had a higher prevalence of depressive symptoms than Belgian or EU citizens [2]. Missinne and Bracke also confirmed the higher depression scores among first and second-generation immigrants in the early 2000s but the authors did not study the evolution of this risk over time [14]. Another study indicated differential access to substance use treatment services for migrants and ethnic minorities in Belgium, consequently amplifying the disparities in mental health [15].

Mental health remains an important public health issue in Belgium. The Belgian Health Interview Survey (BHIS) of 2018 reported that 18.3% of the general population experienced psychological distress. However, there is still little research on mental health prevalence among those from different migration and ethnic backgrounds due to inadequate system of population-based study registration [16, 17]. A prior study utilizing the BHIS to investigate positive mental health based on migration background discovered that individuals without a migration background exhibited higher scores of positive mental health, compared to those with a migration background. The authors also noted that the association between mental health and migration background disappeared when controlling for sociodemographic factors. However, this study did not address the temporal evolution of these findings [18].

Considering the results of previous studies and the lack of information on the long-term evolution of the mental health status among MEM in Belgium, there is a need to pay more attention to the mental health of MEM populations and, to describe their mental health status over time. Despite the growing diversity of the Belgian population, research on mental health status and its evolution across the years is lacking. Conducting research in this area can help fill this gap and provide much-needed insights into the mental health of these important growing population groups.

Therefore, the aim of this study is to understand the evolution of mental illness prevalence among different MEM groups and to determine whether MEM status remains an important factor in mental health status when other risk factors, such as socioeconomic status and parents’ country of birth are controlled for. In summary, this study aims to address the following research questions: How has the prevalence of MI changed over time among different migrant and ethnic minority populations compared to the host country population, and which determinants influence this prevalence?

## Methods

### Data

We pooled data from the six waves of the Belgian Health Interview Survey (BHIS) which is a repeated crossed-sectional epidemiological survey conducted throughout Belgium from 1997 to 2018 (1997, 2001, 2004, 2008, 2013, and 2018). The survey’s purpose is to provide information on the physical and mental health status of the general population and the use of healthcare and services. The target population included all people residing in Belgium, including people with a migration background, regardless of their country of birth or their nationality. The BHIS is representative of the population in private and collective households. People who stayed in psychiatric care or any institutional housing during the time of the interview were not surveyed. All questionnaires were available in French, Dutch, German (the three national languages), and English (for foreigners). When necessary, a translator, who was generally a member of the household who speaks both languages, was used. The response rate to the different surveys was around 60% with a total sample of approximately 10,000 respondents in each wave. Full details of the methodology have been described elsewhere [19, 20].

We included data on mental health for adults aged between 15 and 65 years old because of the small sample of elderly immigrants. We did not include people with missing data for mental health status, who represented less than 5% in each wave. The final sample ranged from 4363 to 6362 subjects in each wave, stratified into five major and minor ethnic groups.

### Migration and ethnic background

The main independent variable in this study was the migration and ethnic background of the respondents, hereafter called ethnicity. In the 1970s, Belgium experienced a significant wave of labour immigration. Later, in the 1990s, the country changed its legislation on naturalization, and nationality became a less reliable indicator of migration and ethnic background. Additionally, migrant descendants automatically acquired Belgian nationality at birth, although their migration and ethnic background may not differ from that of their migrant parents. To capture the maximum amount of information about the respondents’ migration and ethnic background, we decided to calculate a variable called “ethnicity” based on their country of birth or nationality. Administrative registration of ethnicity is not mandatory, and gathering such data remains a sensitive issue in Belgium [21]. Furthermore, the Law on the protection of privacy governs the collection of personal data, allowing it only when is well-defined, clear, and legitimate [22]. Since the primary aim of the BHIS does not revolve around investigating *ethnic* health disparities, our analyses are grounded in the country of birth or nationality instead of ethnicity. For instance, a Belgian born abroad was considered to have a foreign ethnic background, and a foreigner born in Belgium was also considered to have a foreign ethnic background. Having Belgian nationality does not necessarily mean that individuals were not exposed to a higher risk of discrimination, social exclusion, or even racism in their mental health care. Starting in 2013, the health survey included questions about the mother’s and father’s country of birth. Respondents were given three options to choose from (1) Belgium, (2) a European country, or (3) a non-European country. We included this independent variable in the analyses on the data collected in 2013 and 2018.

Based on previous studies that have highlighted people with a Moroccan or Turkish background as having a higher mental health risk than the Belgian population and being considered the groups most at risk of poor health [1, 23], we decided to divide the sample into five major ethnic groups as follows: Belgian, Moroccan, Turkish, European migrants and Non-European migrants [2]. We also based our classification on the availability of the data in the BHIS.

### Mental health status

Mental health status was the dependent variable and was assessed using the 12-item General Health Questionnaire (GHQ-12), which is a widely used screening instrument for psychological distress [24]. The GHQ-12 measures four subscales: somatic symptoms, anxiety and insomnia, social dysfunction, and depression, based on the respondent’s experience in the last four weeks compared to their usual state, using categories such as better, worse, much worse. The GHQ-12 has good psychometric properties, widely used in health surveys, with a Cronbach’s α = 0.90 [24], and the final score ranges from 0 (no psychological distress) to 12 (severe psychological distress). We reported the GHQ-12 score both as a mean to estimate psychological distress severity and as a dichotomous variable using a validated cut-off point of GHQ-score > = 4 to estimate the prevalence of non-psychotic mental illness (MI) [24].

### Other determinants

Other socioeconomic determinants were collected, including sex (man or woman), age (divided into three groups: 15–24, 25–44, and 45–65 years old), ethnicity (classified as Belgian, Moroccan, Turkish, European migrants, and non-European migrants), and level of education (classified as 1 = No diploma or primary education, 2 = Lower or higher secondary, and 3 = Higher or tertiary education). For 2013 and 2018, as mentioned previously, we included the mother’s country of birth and the father’s country of birth variables in the model as questions about these were added to the survey.

### Statistical analysis

Descriptive statistics were computed for socio-demographic characteristics such as sex, age, and ethnicity of respondents and their parents throughout the different survey years. We also presented the GHQ-12 scores (means and SD) and the prevalence of MI (GHQ > = 4) for each survey year. In order to describe the ethnic discrepancies in MI and its evolution across time, a Chi² test and an analysis of variance (ANOVA) were performed using the GHQ-12 score. As a statistically significant difference does not mean that the difference is clinically observable, the minimum clinically important difference (MID) was computed for the GHQ-12 as ${\sigma }_{x}*\sqrt{1-{r}_{x}}$, where “r” is the reliability of the measure. We used the Cronbach’s α of 0.90 [24].

In order to compare mental health risk between ethnic groups and over time, we used a linear regression model to estimate the average marginal effect of ethnicity on GHQ-12 scores, while controlling for age, sex, and level of education. The independent variable was ethnicity, which we classified into five groups. Years of the survey were included as a covariate to account for any changes in the GHQ-12 scores over time. We reported the average marginal GHQ-12 scores (CI95%), for each survey year and ethnic group.

Next, we conducted four linear regression analyses to determine the factors that contribute to mental illness among participants across time. The dependent variable was the GHQ-12 score, which we used as a continuous measure of psychological distress. The independent variables in both models included age, sex, ethnicity, education level, and year. For data from 2013 to 2018, parents’ country of birth was also included as a variable. While the first two models used 1997 as the reference year, the third and fourth models used 2013 as the reference year because they compared psychological distress including parents’ country of birth. The objective of the first and second models was to determine whether ethnicity remained a significant factor in psychological distress when controlling for other socioeconomic factors. The objective of the third and fourth models was to investigate whether second-generation immigrants were more at risk for psychological distress than first-generation immigrants.

Statistical significance was set at p-value < 0.05. All the statistical analyses were performed in SAS® 9.4.

## Results

Table 1 displays the sociodemographic characteristics of the respondents from 1997 to 2018. As mentioned in the methods section above, we only included data on mental health for adults aged between 15 and 65 years old, given the fact that elderly immigrants constituted a small sample among the participants. Therefore, we ended up with a total sample ranging from 4363 to 6362 subjects in each year. Additionally, we know that MEM groups are often underrepresented in national health surveys [8] and, this should be considered when interpreting results. The sample’s distribution was similar over time in terms of age, sex, ethnicity, level of education, and mental health status. However, we observed changes in the ethnic composition of the sample over time. In 1997, 82% of the sample was Belgian, while in 2018, this percentage decreased to 74.9%, which is consistent with the change in population composition [25]. Descriptive statistics showed that the proportion of respondents with a Moroccan background increased from 1.9% in 2001 to 3.2% in 2018, and that of those with a Turkish background increased from 0.9 to 1.1%, respectively. The table also examined the parents’ country of birth of respondents, and the results indicated that the proportion of individuals with one or two parents born abroad increased between 2013 and 2018. For example, the percentage of respondents with a mother born outside the European Union was 11.1% in 2013 but rose to 15.3% in 2018.

**Table 1:** Characteristics of the sample over the years, Belgian Health Interview Survey from 1997 to 2018

	1997 (n = 5771) N (%)	2001 (n = 6362) N (%)	Year 2004 (n = 5943) N (%)	2008 (n = 4845) N (%)	2013 (n = 4363) N (%)	2018 (n = 5334) N (%)
**Sex**						
Man	2869 (49.7)	3129 (49.2)	2845 (47.9)	2332 (48.1)	2081 (47.7)	2540 (47.6)
Woman	2902 (50.3)	3233 (50.8)	3098 (52.1)	2513 (51.9)	2282 (52.3)	2794 (52.4)
**Age**						
15–24	414 (7.2)	401 (6.3)	400 (6.7)	352 (7.3)	188 (4.3)	200 (3.7)
25–44	3020 (52.3)	3192 (50.2)	2790 (46.9)	2173 (44.9)	1912 (43.8)	2229 (41.8)
45–65	2337 (40.5)	2769 (43.5)	2753 (46.3)	2320 (47.9)	2263 (51.9)	2905 (54.5)
**Ethnicity**						
Belgium	4734 (82.0)	5436 (85.4)	4982 (83.8)	3903 (80.6)	3475 (79.6)	3995 (74.9)
Morocco	191 (3.3)	121 (1.9)	130 (2.2)	136 (2.8)	142 (3.3)	171 (3.2)
Turkey	54 (0.9)	60 (0.9)	50 (0.8)	44 (0.9)	39 (0.9)	61 (1.1)
EU country	621 (10.8)	570 (9.0)	582 (9.8)	545 (11.2)	478 (11.0)	703 (13.2)
Non-EU country	171 (3.0)	175 (2.8)	199 (3.3)	217 (4.5)	229 (5.2)	404 (7.6)
**Education**						
No diploma or primary education	900 (15.6)	895 (14.1)	691 (11.6)	439 (9.1)	334 (7.7)	249 (4.7)
Lower or higher secondary	3063 (53.1)	3421 (53.8)	3141 (52.9)	2506 (51.7)	2233 (51.2)	2560 (48.0)
Higher or tertiary education	1808 (31.3)	2046 (32.2)	2111 (35.5)	1900 (39.2)	1796 (41.2)	2525 (47.3)
**Mother’s country of birth**						
Belgium	NA	NA	NA	NA	3306 (75.9)	3737 (70.3)
EU country					565 (13.0)	765 (14.4)
Non-EU country					485 (11.1)	815 (15.3)
**Father’s country of birth**						
Belgium	NA	NA	NA	NA	3270 (75.1)	3728 (70.1)
EU country					584 (13.4)	766 (14.4)
Non-EU country					502 (11.5)	823 (15.5)
**Score GHQ-12** mean (SD)	1.7 (2.7)	1.4 (2.5)	1.4 (2.5)	1.5 (2.6)	1.9 (2.9)	1.9 (2.9)
**Proportion with MI (GHQ > = 4)**	1043 (18.1)	877 (13.8)	825 (13.9)	741 (15.3)	875 (20.1)	1057 (19.8)

It is noticeable that the proportion of MI (GHQ score of 4 or higher) has changed over time, increasing from 13.8% in 2001 to 19.8% in 2018.

**Fig. 1:**
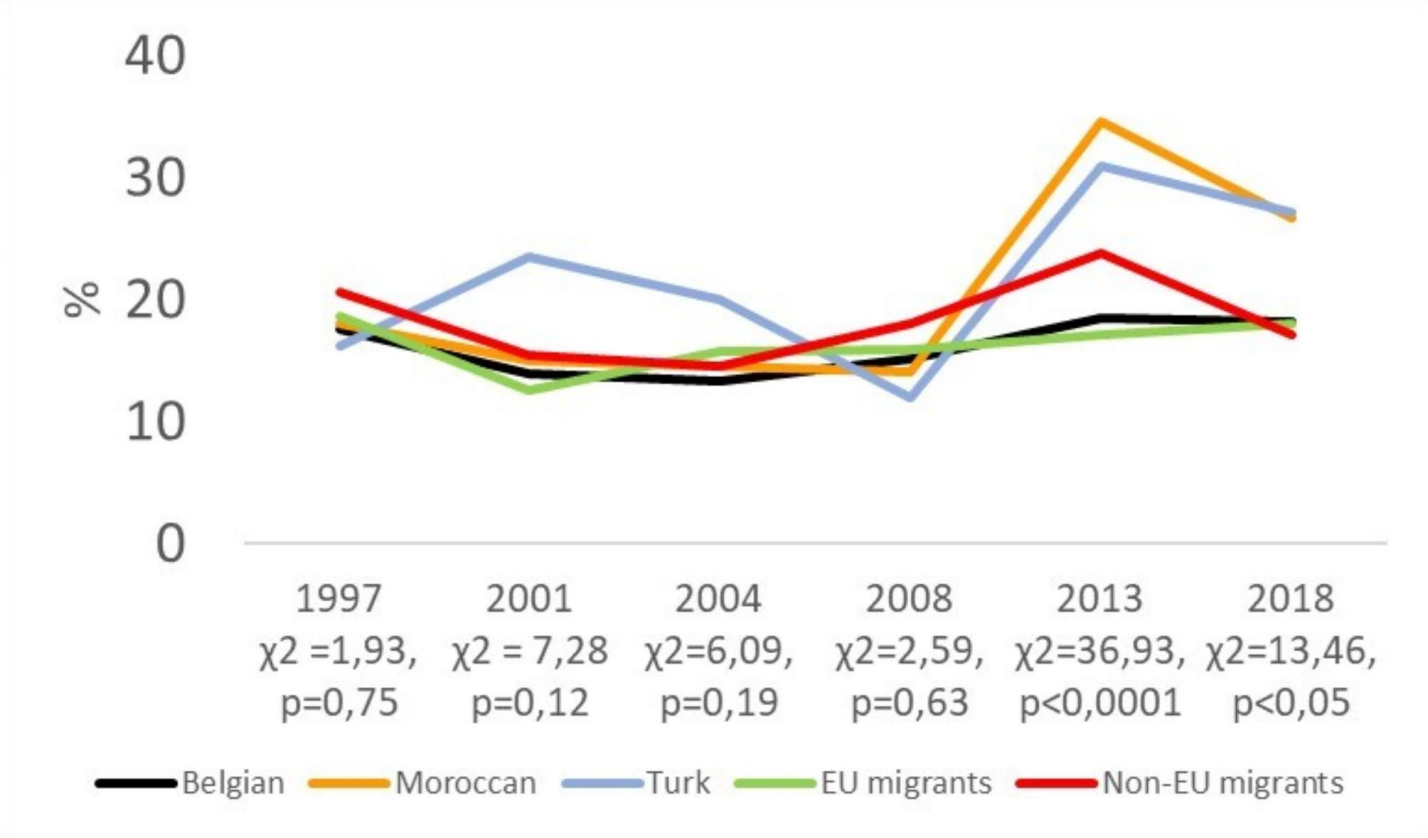
Prevalence of mental illness (GHQ ≥ 4) by ethnic group and by year

Figure 1 shows that the proportion of people with MI was not significantly different among ethnic groups in 1997, 2001, 2004, and 2008. However, there was a significant difference observed in 2013 and 2018. Specifically, in 2013, there was a steady increase in the proportion of MI among Belgian respondents and an even greater increase among respondents with a Moroccan or Turkish background. In 2018, the proportion of MI slightly decreased among all ethnic groups, but the crude prevalence of MI remained higher among respondents with a Moroccan or Turkish background.

Table 2 presents the average marginal mean of psychological distress (GHQ-score) from the linear regression, for each of the five ethnic groups, for each year, as well as the Minimal Important Difference (MID). The scores were adjusted for age, sex, and education level of participants. The findings indicated that, on average, the score of mental distress has increased between 1997 and 2018 for all ethnic groups compared to Belgians, except for non-EU migrants. More specifically, there was a significant increase in the score for respondents with a Moroccan or Turkish background between 2008 and 2018, while the score remained stable for the other groups. In other words, the mental health of Moroccans and Turks did not differ from that of Belgians in each survey wave. However, over time, the mental health of Moroccans and Turks deteriorated in comparison to that of Belgians.

**Table 2:** Evolution of psychological distress (GHQ-12 score) in five ethnic groups in Belgium from 1997 to 2018, with average marginal effect controlled for age, sex, and education

	GHQ-12 score Average marginal mean (CI95%)^a^						Chi²-test^b^ (p-value)
	1997	2001	2004	2008	2013	2018	
**Belgium**	1.65 (1.58;1.72)	1.35 (1.29;1.41)	1.34 (1.28;1.40)	1.49 (1.42;1.56)	1.81 (1.74;1.81)	1.87 (1.77;1.97)	Ref
**Morocco**	1.61 (1.25;1.97)	1.44 (0.99;1.89)	1.41 (0.97;1.84)	1.42 (0.99;1.86)	3.19 (2.78;3.61)	2.50 (2.10;2.91)	**17.75 (< 0.001)**
**Turkey**	1.39 (1.69;2.09)	1.86 (1.21;2.51)	1.50 (1.20;2.20)	1.11 (0.37;1.85)	2.93 (2.12;3.74)	2.49 (1.81;3.17)	**4.19 (0.04)**
**EU migrants**	1.70 (1.51;1.88)	1.23 (1.04;1.42)	1.50 (1.02;1.69)	1.51 (1.32;1.71)	1.62 (1.41;1.84)	1.80 (1.60;1.99)	1.06 (0.30)
**Non-EU migrants**	1.85 (1.46;2.23)	1.42 (1.05;1.79)	1.52 (1.08;1.87)	1.64 (1.30;1.98)	2.38 (2.06;2.71)	1.75 (1.48;2.02)	0.06 (0.80)
**MID** ^**c**^	0.85	0.77	0.77	0.81	0.90	0.89	

Each year estimates are computed from the same model

^a^ Average marginal mean computed from the linear regression model controlled for age, sex, and education

^b^ Wald Chi² from the linear regression model, with variable “year” was considered as a continuous variable

^c^ The Minimal Important Difference (MID) is calculated as σ_x*√(1-r_x )

The results of the MID also indicate that respondents with a Turkish background had a clinically higher score of mental distress than Belgians in three years out of the six (2001, 2013, and 2018). Similarly, people with a Moroccan background had clinically higher scores in 2013 and 2018.

Table 3 displays the estimates (β, CI95%) obtained from linear regression analyses assessing the determinants of psychological distress severity among the study participants. All the models were controlled for the survey year. The first model presents the estimates for the main effect of ethnicity. The second model is controlled for the socioeconomic status (SES) of respondents. The third model presents the main effect of the parents’ country of birth on psychological distress while SES was not included. The last model includes the parents’ country of birth while controlling for SES. In the first two models, we found that ethnicity had no significant impact on psychological distress severity, even when not accounting for socioeconomic factors such as age, sex, and education. Models 3 and 4 indicated that after adjusting for socioeconomic status, non-EU migrants were less vulnerable to psychological distress than Belgians in 2018 (β=-0.55, CI95%=-0.90; -0.19). Another noteworthy finding was that having a mother born outside the EU initially acted as a protective factor against psychological distress, but became a risk factor after controlling for socioeconomic factors, in 2018. This suggests that second-generation migrants faced a higher risk of mental illness compared to Belgians. However, the country of birth of the father did not appear to affect psychological distress levels.

**Table 3:** Linear regression estimates* (β, CI95%) for factors influencing psychological distress (GHQ-12 score) among adults aged 15–65 since 1997

	Model 1			Model 2				Model 3 (Data from 2013 to 2018)		Model 4 (Data from 2013 to 2018)	
**Ethnicity**	Yes			Yes				Yes		Yes	
**SES (age, sex, education)**	No			Yes				No		Yes	
**Parents’ country of birth**	No			No				Yes		Yes	
**Ethnicity (Ref = Belgian)**											
Morocco	0.10	(-0.24;0.44)		-0.06		(-0.45;0.33)		0.50	(0.12;0.88)	0.06	(-0.39;0.51)
Turkey	-0.26	(-0.91;0.39)		-0.26		(-0.97;0.46)		0.39	(-0.18;0.97)	-0.14	(-0.79;0.51)
EU country	0.10	(-0.09;0.29)		0.05		(-0.17;0.27)		-0.20	(-0.44;0.03)	-0.28	(-0.57;0.01)
Non-Eu country	0.21	(-0.13;0.55)		0.13		(-0.28;0.53)		-0.31	(-0.60;-0.01)	**-0.55**	**(-0.90;-0.19)**
**Mother’s country of birth (Ref = Belgium) for 2018 (Ref = 2013)**											
EU country								0.02	(-0.30;0.34)	-0.01	(-0.40;0.37)
Non-EU country								**0.84**	**(0.30;1.37)**	**1.38**	**(0.71;2.05)**
**Father’s country of birth (Ref = Belgium)**											
EU country								0.07	( -0.25;0.39)	0.12	(-0.26;0.50)
Non-EU country								-0.12	( -0.65;0.41)	-0.31	(-0.96 ;0.34)

*Controlled for years

## Discussion

### Main results

The purpose of this study was to investigate ethnic differences in mental health and its determinants among adult populations in Belgium from 1997 to 2018, with a focus on ethnic differences. Results showed that the prevalence of MI changed over time. Overall, the prevalence of MI increased between 1997 and 2018 among all migrant and ethnic groups, except for the non-EU migrants, for whom the prevalence slightly decreased in 2018. Between 2013 and 2018, the prevalence of MI slightly decreased but remained higher among respondents with a Moroccan or Turkish background, compared to Belgians. Linear regression analysis highlighted the main effect of ethnicity on mental illness was not significant, even when socioeconomic factors were controlled. However, in 2018, being of non-EU country descent was associated with a decreased risk of psychological distress when the parents’ country of birth was accounted for. Interestingly, in the same year, it was found that having a mother born outside the EU constituted a higher risk of psychological distress.

Overall, the result suggested that there were ethnic disparities in the prevalence of mental illness in the population surveyed in Belgium. Specifically, the proportion of individuals with MI was not significantly different among MEM groups in earlier years, from 1997 to 2008, but became significantly different in more recent years, with a greater increase observed among respondents with a Moroccan or Turkish background. This result was in line with national and international epidemiology for psychopathology in Moroccan and Turkish descents [2, 26]. However, it was found that socioeconomic differences explained the major ethnic differences in MI prevalence over the years. This result was also concordant with a previous study based on the third European Social Survey [14].

It is worth noting that MEM currently account for around 20% of Belgium’s population, with the largest groups hailing from Morocco, Turkey, and Italy [25]. Despite this, immigration remains a politically sensitive issue in Belgium, and debates surrounding integration, social cohesion, and the economic impact of immigration continue to dominate public discourse. Looking back to the 1960s, Belgium experienced a significant wave of migration following the signing of bilateral agreements with countries like Turkey, Morocco, and Tunisia to recruit labour workers. Many of these immigrants ended up staying in Belgium and brought over their families, leading to an increase in the immigrant population. In the 2000s, there was a rise in the ratio of Moroccan citizens entering the country compared to other foreigners, increasing from 4.5 to 10.7% after the 1999 regularization campaign. However, since 2004, this percentage has decreased, with Moroccan immigration accounting for only 5% of foreign entries in 2012.

In 2008, Belgium experienced an economic recession which intensified the economic exclusion and unemployment of people with MI, such as ethnic minorities, compared to the general population [27]. Moreover, negative attitudes towards migrant and ethnic minority groups could have been exacerbated during economic hardship, potentially impacting their mental health status [27]. Previous research has indicated that individuals facing stigma related to their mental health tend to have worse life chances than those without mental illness [28]. During a crisis, individuals may also experience a lack of belonging to Belgian society, further impacting their mental health. Studies have shown a correlation between acculturation and mental health in other contexts [29], and these results might help explain the significant increase in MI prevalence among respondents with a Moroccan or Turkish migration background in 2013.

A previous study highlighted that second-generation migrants had a higher MI risk than first-generation [14], which is consistent with our own findings in 2018, showing that having a mother born outside European countries increased the risk of MI. The mental health of parents may also play a role in influencing their children’s (mental) health, as demonstrated in other contexts [30]. Another potential explanation for this increased risk among people with a second-generation migration background is that they often experience an identity conflict, as they attempt to navigate between the culture of their parents and the culture of the society they grew up in [31]. This struggle can lead to anxiety and depression, which may increase the likelihood of mental health problems [31]. Additionally, people with a second-generation migration background may face discrimination and prejudice due to their migration or ethnic background in various domains of life, including education, labour markets, housing, and health care, which can also contribute to poorer mental health outcomes [1].

However, there is not enough data to assess the evolution of MI prevalence among second-generation MEM since 1997. Therefore, it is recommended that future population health surveys collect data on the country of birth of the father and mother more precisely to describe the evolution of the mental health status of people with a second-generation migration background. Further, this recommendation is in line with a previous article that recommended “to add additional purposive sampling strategies to the National Health Surveys in order to routinely collect data in representative MEM samples” in the European context [32]. This could help to adapt healthcare to specific ethnic groups, including second-generation migrants, which represent an important migrant and ethnic group in Belgium.

Researcher has noted the underrepresentation of ethnic minority groups in national health surveys [8]. In our study, the ethnic minority groups ranged from 0.7% of the sample, represented by the EU migrants in 2004, to 13.4% of non-EU migrants in 2018, which was not comparable to the 30.2% of the Belgian population with a foreign country of birth [25]. This underrepresentation leads to underpowered samples and non-statistically significant conclusions in research on health ethnic inequalities [33]. The underrepresentation could be due to language barriers or the lack of instruments available in the respondent’s language [8, 14]. Although the study’s language was available in the three national languages and English for foreigners, we could not determine whether non-language speakers were adequately assisted in translating the survey. Another possible explanation is a cultural bias in their GHQ-12-items answers, which may not reflect their actual mental health status. This underrepresentation of migrants and ethnic minorities in mental health surveys has led to rare and patchy mental health disorder prevalence studies among them, according to several studies [1, 8, 14]. We would recommend translating large health interview surveys into other languages for future research to obtain better samples of migrant and ethnic minority populations.

### Strengths and limitations

One strength of this study is that it provides the first nationwide community-based results on the evolution of mental health risk prevalence and its determinants in important ethnic groups in Belgium. Another strength main is the use of six cross-sectional data-collection waves. These data allowed us both to assess the evolution over the past 20 years and to compare different ethnic groups with native Belgians.

One limitation of this study is that ethnicity was not self-reported as it is commonly the case in other countries, but instead computed a variable based on respondents’ country of birth and nationality. In order to obtain more sensitive and accurate information regarding respondents’ ethnicity, we recommend that future research include a self-reported ethnicity question in health interview surveys. This approach is recommended by guidelines such as those provided by the National Institutes of Health in the United States [34] and by the National Health Service in the United Kingdom [35]. Indeed, as shown in our results, ethnicity can be an important factor in determining mental health outcomes and access to healthcare services. By collecting these data on self-reported ethnicity, researchers and policymakers could better identify and address health ethnic disparities among different ethnic groups in Belgium. Further, self-reported ethnicity can provide insight into cultural factors such as language, religion, and customs that might impact mental health. Therefore, including self-reported ethnicity questions in the health survey can be used to inform public health policy ensuring that health care is distributed equitably among different MEM groups. Indeed, it is unacceptable that different ethnic groups continue to receive unequal care nowadays.

Another limitation is the composition of our sample, as previously mentioned. The subgroup of ethnic minorities and migrants included in the study may be not representative of the immigrant population in Belgium. The Belgian Health Interview Survey does not allow us to measure ethnic inequalities in mental health. To remedy this, we could oversample certain migrant and ethnic groups in health interview surveys to ensure that enough representative data is collected regarding their mental health.

Another limitation could be the decision to group people from non-European countries into one common ethnic group, as their ethnic background and socioeconomic status may vary and influence their mental health status. However, this decision was made to ensure sufficient statistical power for our analyses. To address this limitation, we recommend collecting more precise data on participants’ country of birth to enable analysis of each ethnic subgroup separately. Nonetheless, this study is the first to describe the evolution of MI prevalence among different ethnic groups in Belgium and provides insights into ethnic disparities in mental health over time.

## Conclusions

In conclusion, it is clear that people with a Moroccan or Turkish migration background in Belgium face significant persisting mental health disparities compared to Belgians. This study has also shown that these groups have a higher risk of MI compared to Belgians and that these disparities have increased since 2013. It is imperative to collect more accurate data on ethnic origin in large health interview surveys and develop targeted interventions and policies to support the mental health of ethnic minorities and migrants in Belgium. By doing so, we can work towards reducing these ethnic disparities and promoting better overall (mental) health outcomes for all ethnic groups in the country.

## Data Availability

The data that support the findings of this study are available from Sciensano but restrictions apply to the availability of these data, which were used under license for the current study, and so are not publicly available. Data are however available from the authors upon reasonable request and with permission of Sciensano.

## Abbreviations

BHIS: Belgian Health Interview Survey
MEM: Migrant(s) and Ethnic minority(ies)
MI: Mental Illness
