# Correction: Mental health among people with a migration background in Belgium over the past 20 years: how has the situation evolved?

**DOI:** 10.1186/s13690-023-01199-9

**Published:** 2023-11-21

**Authors:** Camille Duveau, Pierre Smith, Vincent Lorant

**Affiliations:** 1 Faculty of Public Health, Institute of Health and Society (IRSS), Université catholique de Louvain, B1.31.15, Brussels, 1200 Belgium; 2 Department of Epidemiology and Public Health, Sciensano, Brussels, Belgium

**Correction to**: *Archives of Public Health*** (2023) 81:176**

10.1186/s13690-023-01187-z

Following publication of the original article [1], the authors found three errors with the authorship, Table 1 and related content in the Results section. Details are as follows:

- The given and family names of each author were published in reverse. The authorship panel therefore read: Duveau Camille, Smith Pierre, and Lorant Vincent.
- Table 1 needs to be updated, it can be found below.
- The sentences that describe Table 1 in the **Results** section need to be updated. The changes have been highlighted in bold typeface.
  “In 1997, **82.0%** of the sample was Belgian, while in 2018, this percentage decreased to **74.9%**, which is consistent with the change in population composition [25]. Descriptive statistics showed that the proportion of respondents with a Moroccan background increased from **1.9%** in 2001 to **3.2%** in 2018, and that of those with a Turkish background increased from **0.9 to 1.1%**. The table also examined the parents’ country of birth of respondents, and the results indicated that the proportion of individuals with one or two parents born abroad increased between 2013 and 2018. For example, the percentage of respondents with a mother born outside the European Union was 11.1% in 2013 but rose to 15.3% in 2018.
  It is noticeable that the proportion of MI (GHQ score of 4 or higher) has changed over time, increasing from **13.8%** in 2001 to **19.8%** in 2018.”

The original article [1] has been corrected.

**Table 1:** Characteristics of the sample over the years, Belgian Health Interview Survey from 1997 to 2018

	Year								
	1997 (n = 5771)	2001 (n = 6362)	2004 (n = 5943)		2008 (n = 4845)		2013 (n = 4363)		2018 (n = 5334)
	N (%)	N (%)	N (%)		N (%)		N (%)		N (%)
**Sex**									
Man	2869 (49.7)	3129 (49.2)	2845 (47.9)		2332 (48.1)		2081 (47.7)		2540 (47.6)
Woman	2902 (50.3)	3233 (50.8)	3098 (52.1)		2513 (51.9)		2282 (52.3)		2794 (52.4)
**Age**									
15–24	414 (7.2)	401 (6.3)	400 (6.7)		352 (7.3)		188 (4.3)		200 (3.7)
25–44	3020 (52.3)	3192 (50.2)	2790 (46.9)		2173 (44.9)		1912 (43.8)		2229 (41.8)
45–65	2337 (40.5)	2769 (43.5)	2753 (46.3)		2320 (47.9)		2263 (51.9)		2905 (54.5)
**Ethnicity**									
Belgium	4734 (82.0)	5436 (85.4)	4982 (83.8)		3903 (80.6)		3475 (79.6)		3995 (74.9)
Morocco	191 (3.3)	121 (1.9)	130 (2.2)		136 (2.8)		142 (3.3)		171 (3.2)
Turkey	54 (0.9)	60 (0.9)	50 (0.8)		44 (0.9)		39 (0.9)		61 (1.1)
EU country	621 (10.8)	570 (9.0)	582 (9.8)		545 (11.2)		478 (11.0)		703 (13.2)
Non-EU country	171 (3.0)	175 (2.8)	199 (3.3)		217 (4.5)		229 (5.2)		404 (7.6)
**Education**									
No diploma or primary education	900 (15.6)	895 (14.1)	691 (11.6)		439 (9.1)		334 (7.7)		249 (4.7)
Lower or higher secondary	3063 (53.1)	3421 (53.8)	3141 (52.9)		2506 (51.7)		2233 (51.2)		2560 (48.0)
Higher or tertiary education	1808 (31.3)	2046 (32.2)	2111 (35.5)		1900 (39.2)		1796 (41.2)		2525 (47.3)
**Mother’s country of birth**									
Belgium	NA	NA	NA		NA		3306 (75.9)		3737 (70.3)
EU country							565 (13.0)		765 (14.4)
Non-EU country							485 (11.1)		815 (15.3)
**Father’s country of birth**									
Belgium	NA	NA		NA		NA		3270 (75.1)	3728 (70.1)
EU country								584 (13.4)	766 (14.4)
Non-EU country								502 (11.5)	823 (15.5)
**Score GHQ-12** mean (SD)	1.7 (2.7)	1.4 (2.5)	1.4 (2.5)		1.5 (2.6)		1.9 (2.9)		1.9 (2.9)
**Proportion with MI (GHQ > = 4)**	1043 (18.1)	877 (13.8)	825 (13.9)		741 (15.3)		875 (20.1)		1057 (19.8)
